# Understanding the Role of SERCA2a Microdomain Remodeling in Heart Failure Induced by Obesity and Type 2 Diabetes

**DOI:** 10.3390/jcdd9050163

**Published:** 2022-05-19

**Authors:** Ping Lai, Viacheslav O. Nikolaev, Kirstie A. De Jong

**Affiliations:** 1 Institute of Experimental Cardiovascular Research, University Medical Center Hamburg-Eppendorf, D-20246 Hamburg, Germany; p.lai.ext@uke.de; 2 German Center for Cardiovascular Research (DZHK), Partner Site Hamburg/Kiel/Lübeck, D-20246 Hamburg, Germany; 3 Department of Cardiology, First Affiliated Hospital of Gannan Medical University, Key Laboratory of Prevention and Treatment of Cardiovascular and Cerebrovascular Diseases, Ministry of Education, Gannan Medical University, Ganzhou 341000, China

**Keywords:** cAMP, PLN, SERCA2a microdomain, type 2 diabetes, obesity, HFpEF

## Abstract

Obesity and type 2 diabetes (T2D) are on trend to become a huge burden across all ages. They cause harm to almost every organ, especially the heart. For decades, the incidence of heart failure with impaired diastolic function (or called heart failure with preserved ejection fraction, HFpEF) has increased sharply. More and more studies have uncovered obesity and T2D to be closely associated with HFpEF. The sarcoplasmic/endoplasmic reticulum calcium ATPase2a (SERCA2a) microdomain is a key regulator of calcium reuptake into the sarcoplasmic reticulum (SR) during diastole. 3′,5′-cyclic adenosine monophosphate (cAMP) and its downstream effector cAMP dependent protein kinase (PKA) act locally within the SERCA2a microdomain to regulate the phosphorylation state of the small regulatory protein phospholamban (PLN), which forms a complex with SERCA2a. When phosphorylated, PLN promotes calcium reuptake into the SR and diastolic cardiac relaxation by disinhibiting SERCA2a pump function. In this review, we will discuss previous studies investigating the PLN/SERCA2a microdomain in obesity and T2D in order to gain a greater understanding of the underlying mechanisms behind obesity- and T2D-induced diastolic dysfunction, with the aim to identify the current state of knowledge and future work that is needed to guide further research in the field.

## 1. Introduction

As the world lives longer, obesity and T2D are becoming more and more frequent all over the world, with the global prevalence of obesity and T2D estimated to reach a staggering 1 billion and 570 million, respectively, by the year 2025 [1]. As obesity and T2D increase the risk for cardiovascular diseases [2], they not only result in a heavy economic burden and medical stress, but also pose a serious impact to quality of life. Epidemic studies have shown obesity and T2D go hand in hand, and contribute to many kinds of cardiovascular diseases, in particular to heart failure with preserved ejection fraction (HFpEF) [3,4]. HFpEF draws our attention due its high morbidity and lack of suitable therapies, particularly in obesity and T2D associated HFpEF [5,6]. In efforts to identify new effective therapies, it is imperative to uncover the role of obesity and T2D in the pathophysiology of HFpEF. Investigations into the PLN/SERCA2a microdomain provide a hopeful spark in achieving this goal [7]. This microdomain is particularly interesting because it controls diastolic function of the cardiomyocyte by regulating calcium cycling [8], with the level of cAMP determining whether the SERCA2a pump is more “opened or closed” by PKA mediated phosphorylation of PLN. In this review, we aim to discuss what is currently known about the PLN/SERCA2a microdomain in the obese and T2D heart in the context of HFpEF, and to highlight any obstacles and limitations that need to be overcome in order to direct this research field and potential new HFpEF therapies in the future.

## 2. Obesity and T2D

Obesity is defined as a disproportionate body weight for height with an excessive accumulation of adipose tissue, and it is assessed via body mass index (BMI), expressed as the ratio of body weight in kilograms divided by height in square meters (kg/m^2^) [9]. The World Health Organization (WHO) classification defines obesity as a BMI ≥ 30 kg/m^2^ and extreme/morbid obesity as a BMI ≥ 40 kg/m^2^ [10]. T2D is characterized by hyperglycemia, as a result of insulin resistance. While early stages are often accompanied by hyperinsulinemia, if left untreated, the condition may progress to a severe stage of insulin deficiency due to pancreatic beta cell dysfunction [11]. The WHO diagnosis criteria for T2D is based on a fasting blood glucose level of ≥7.0 mmol/L or 2 h post-glucose load ≥ 11.1 [12]. Both obesity and T2D result in significant physical and psychological stress. Additionally, importantly, the combined presence of both obesity and T2D increase cardiovascular risk, with obese and T2D patients being 2–5 times more likely to develop heart failure, of which 75% of cases are attributed to HFpEF [2,13,14].

## 3. Heart Failure Induced by Obesity and T2D

HFpEF differs from traditional heart failure with reduced ejection fraction (HFrEF), in that the heart is still able to pump blood out to the body, but it has an impaired ability to adequately relax [13]. Importantly, HFpEF is reported to have comparable morbidity and mortality rates as HFrEF [15]. For example, mortality rates six months post diagnosis have been reported to be at 16% in both HFpEF and HFrEF patients [16], at one year post diagnosis increasing in both forms of heart failure to 28% and 32%, and at five years to 65% and 68%, respectively [17]. It is also estimated in the latest epidemiological studies that half of all heart failure patients have HFpEF [18]. With obesity and T2D incidence increasing, and with a multitude of studies showing that obesity and T2D promote HFpEF [3,4,19], even in children [20], it is not surprising that HFpEF will soon take over as the predominant form of heart failure [21]. When we consider morbidity and mortality rates associated with HFpEF, it is important to note that in addition to obesity and T2D, there is also a high prevalence of cardiovascular comorbidities that associate with HFpEF, most notably hypertension, coronary artery disease and atrial fibrillation [16]. The presence of these co-morbidities not only contribute significantly to the overall burden of HFpEF but add to the complexity in our understanding of its pathophysiology. Indeed, despite a plethora of potential mechanisms of HFpEF being identified, such as chronic inflammation, hypertension, endothelial cell dysfunction, and dyslipidemia [22,23,24], we still lack significant understanding of how obesity and T2D induce HFpEF. In addition, while prognosis of traditional HFrEF has improved as a result of a variety of effective drugs such as beta blockers, angiotensin-converting enzyme inhibitors and aldosterone antagonists, they are unfortunately ineffective in HFpEF patients [18,25,26,27], particularly in obesity and T2D associated HFpEF. Therefore, we have an urgent need to identify the mechanism/s of HFpEF.

While a growing body of science has nicely focused on the clinical characteristics and diagnosis of HFpEF, including investigations into potential biomarkers [13,28,29], it is the investigation of HFpEF at the cell signaling level that is required for us to truly dissect the pathophysiology of HFpEF. In past work, the lack of suitable HFpEF animal models that are comparable to the clinical manifestation of obesity and T2D HFpEF has been a major impediment. In this review, in an attempt to unravel the mystery of obesity and T2D-induced HFpEF, we rely on past work that was predominately undertaken in murine models of obesity and T2D [30,31,32,33,34]. It is therefore important to note that these models vary considerably in the severity and stage of both obesity and T2D; as such, care must be taken when making comparisons between past work. In addition, while obesity and T2D are strong promoters of HFpEF, they are not the only causes, with increasing age and hypertension in particular, also shown to promote HFpEF. Additionally, indeed, a combination of all of these stresses is often presented in the clinical manifestation of HFpEF. However, in this review, we specifically focus on cAMP dynamics within the PLN/SERCA2a microdomain in the etiology of HFpEF induced by obesity and T2D.

## 4. cAMP and Its Microdomains

cAMP is a ubiquitous intracellular second messenger that regulates a wide range of physiological and pathological processes through the phosphorylation of several proteins involved in calcium handling and excitation-contraction coupling mediated via cAMP dependent protein kinase (PKA) activation [35]. The distinct function of cAMP is dependent on its localization within microdomains (or nanodomains) which are a series of local macromolecular signaling complexes that control the production, regulation, and function of cAMP. For example, cAMP activity is compartmentalized within microdomains situated at the PLN/SERCA2a complex, ryanodine receptors (RyRs) and L-type calcium channel (LTCC), in which increases in cAMP lead to increased PKA activation and subsequent activation of these calcium handling proteins [36]. cAMP production is stimulated via beta-adrenergic receptor (β-AR) signaling, in response to the binding of hormones and neurotransmitters such as epinephrine or norepinephrine to the β_1_-AR or β_2_-AR subtypes. Agonist binding to both the β_1_-AR and β_2_-AR allows for coupling with a stimulatory G protein, Gα_s_, thus stimulating cAMP production. In addition, prolonged agonist binding to the β_2_-AR allows for coupling with an inhibitory G protein, Gα_i_, thus inhibiting cAMP production. cAMP is also inhibited via hydrolysis by phosphodiesterases (PDEs) for which there are at least five isoforms expressed in the heart: PDE1, PDE2, PDE3, PDE4 and PDE8 [36,37]. To prevent sustained stimulation of β-ARs, a negative feedback loop is initiated, in which β-ARs are phosphorylated by G protein-coupled receptor kinases and PKA to promote receptor desensitization [38]. Furthermore, PKA can phosphorylate several cAMP degrading PDE isoforms further enhancing their hydrolytic activity. This dynamic balance of cAMP production and inhibition is vital in achieving distinct functional activities of cAMP within different microdomains [36].

**Figure 1:**
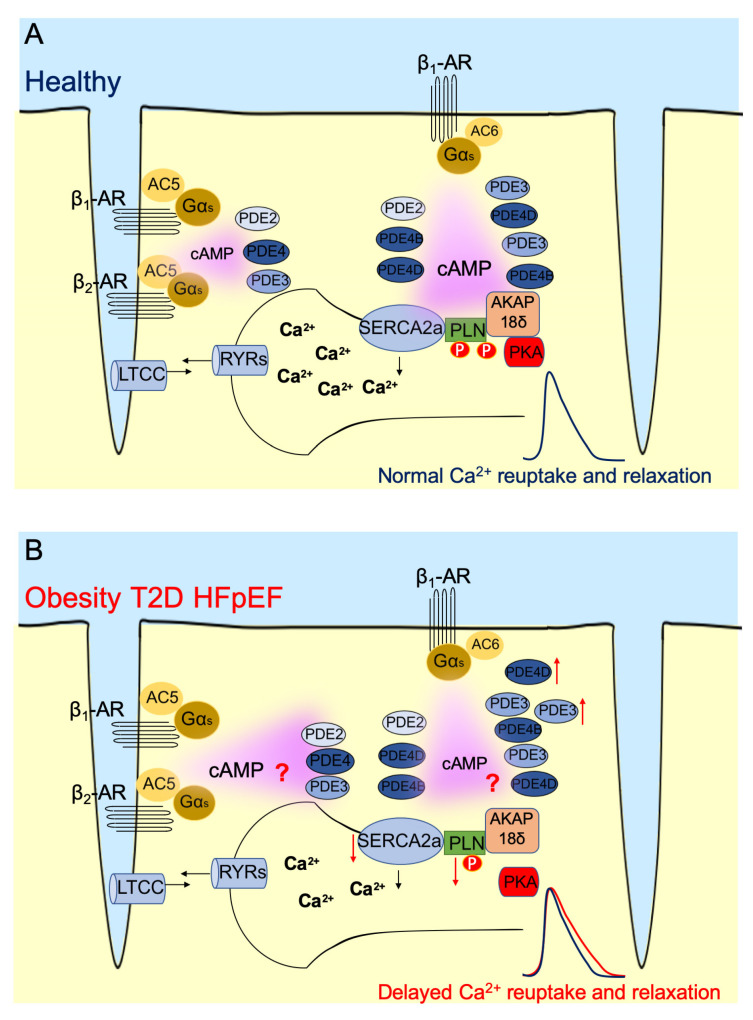
Schematic of the PLN/SERCA2A microdomain in healthy and diseased cardiomyocytes. (**A**) In healthy cells, the regulation of cAMP within the beta-adrenergic receptor (β-AR) microdomains in which cAMP signals stimulated via the β_1_-AR are far-reaching and regulated via the phosphodiesterases (PDEs), PDE2, PDE3 and PDE4, while the β_2_-AR signals are locally confined and regulated via PDE3 and PDE4. In the PLN/SERCA2a microdomain, PKA is tethered by A-kinase anchoring protein 18δ (AKAP18δ) and cAMP pools are mainly regulated via PDE3 and PDE4. (**B**) Schematics of PLN/SERCA2A microdomain remodeling in obesity and T2D-induced HFpEF cardiomyocytes. While there are consistent reports of reduced SERCA2a activity, the mechanisms remain unclear. With typically reduced PLN phosphorylation there is no clear knowledge regarding cAMP concentrations or PDE activities within this microdomain which may lead to spatial alterations in β_1_-AR and β_1_-AR/cAMP responses.

While the majority of past work has been restricted to assessing cAMP dynamics at the whole tissue/cell level with basic biochemical approaches, the development of Förster resonance imaging transfer (FRET) cAMP biosensors specifically targeted to different cAMP microdomains, have allowed the direct assessment of cAMP dynamics within functionally relevant sites of calcium handling with high spatial and temporal resolution [39,40]. Incredibly, this work can be performed in single live cells, allowing us to not only visualize cAMP levels, but to manipulate and measure changes in cAMP concentrations in real time. For obesity/T2D-induced HFpEF, the PLN/SERCA2a microdomain should be of particular functional relevance since it regulates diastolic calcium reuptake and cell relaxation. Live cell imaging using biosensors targeted to this microdomain can potentially provide important new mechanistic insights which are almost completely lacking for HFpEF. For example, we have previously generated FRET-based cAMP biosensor Epac1-cAMP fused to PLN (Epac1-PLN), allowing for the assessment of cAMP dynamics within close proximity to SERCA2a [41]. In this work, we discovered that selective stimulation of the β_1_-AR subtype, but not β_2_-AR, resulted in increased cAMP levels within the PLN/SERCA2a microdomain, and for these cAMP pools to be hydrolyzed by the PDE families in the predominant order of PDE4, PDE3 and PDE2 (see Figure 1). Interestingly, in response to transthoracic-aortic-banding-induced hypertrophy and an early HFrEF phenotype, we observed reduced β-AR-induced cAMP pools within the SERCA2a microdomain and an increased coupling of PDE2. This suggests that in traditional heart failure there is an early remodeling of cAMP dynamics that blunt β-AR cAMP signals within the PLN/SERCA2a microdomain that is partly explained by increased PDE2 mediated hydrolysis of cAMP. However, whether and how cAMP remodeling within the PLN/SERCA2a microdomain occurs in HFpEF is still unclear. Below, we discuss the importance of the PLN/SERCA2a microdomain and evidence obtained from basic biochemical approaches that suggest the presence of PLN/SERCA2a dysregulation and remodeling of cAMP dynamics in obesity and T2D HFpEF models.

## 5. PLN/SERCA2a Microdomain in Heart

The PLN/SERCA2a microdomain is situated within the sarcoplasmic reticulum (SR) membrane. The SR is a vital intracellular organelle found in muscle cells such as cardiomyocytes, smooth muscle cells, and skeletal muscle cells [42]. The function of the SR is to synthesize a portion of proteins and to store calcium needed for excitation-contraction coupling. SERCA2a is the only subtype of SR ATPase expressed in cardiomyocytes that participates in calcium transport [43]. During diastole, more than 70% of cytosolic calcium is absorbed by SERCA2a in reuptake [8]. The downregulation or hypofunction of SERCA2a has been associated with impaired function in the cardiomyocyte, especially in HFrEF [44,45]. Conversely, researchers have also discovered that upregulation of SERCA2a in the atrial myocardium of T2D patients is also associated with impaired diastolic function [46], highlighting the layers of complexity in the regulation of SERCA2a activity besides its expression level. SERCA2a activity is tightly regulated by PLN (Figure 1A).

PLN is a small 52 amino acid protein that was first discovered in the 1970s [47]. It quickly became a hotspot of investigation due to its involvement in regulating SERCA2a activity, with subsequent work targeting PLN as potential therapeutic target for ventricle dysfunction [48]. The SR membrane contains both monomer and pentamer PLN, but only the monomer is capable of binding to and inhibiting SERCA2a [8,49]. PLN binds to SERCA2a by crosslinking lysine (Lys) 3 in the PLN domain Ia to Lys397 and Lys400 in the cytosolic nucleotide-binding domain of SERCA2a [50]. On the one hand, the functional stoichiometry of PLN and SERCA2a in the heart is 2:1 to 2.5:1, and either less or more PLN expression in the cardiomyocyte may result in heart dysfunction [51,52,53,54]. PLN association and inhibition of SERCA2a is relieved upon PLN phosphorylation. With PLN phosphorylated by PKA at serine 16 in response to increased cAMP levels and by Ca^2+^/calmodulin dependent protein kinase II (CaMKII) at threonine 17. PLN is dephosphorylated by protein phosphatase 1 [8].

## 6. PLN/SERCA2a Microdomain in Heart Dysfunction Induced by Obesity and T2D

There is evidence to suggest a dysregulation of PLN/SERCA2a activity in the obese and/or T2D heart that may promote diastolic dysfunction and HFpEF, with many models observing a decrease in SERCA2a activity and altered calcium handling; however, the potential mechanisms of this dysregulation have varied and/or remained unclear.

For example, single cell experiments in cardiomyocytes isolated from db/db mice uncovered a delay in calcium transients, with removal of cytosolic calcium ~33% slower in db/db cardiomyocytes compared to that in healthy mice [55]. The cardiomyocytes also exhibited increased SR calcium leakage. Since this was not accompanied by changes in RyR or sodium-calcium exchanger activity, the authors hypothesized that delayed calcium transients and leakage were due to impaired SERCA2a activity. Indeed, they also observed a threefold greater PLN:SERCA2a expression ratio which would favor an increased inhibitory effect of PLN on SERCA2a. Interestingly, this altered expression ratio was less driven by a reduction in SERCA2a expression, but more so due to a large increase in PLN expression. Accompanying this altered expression ratio, the db/db cardiomyocytes also exhibited a trend for a reduced PLN Ser16 phosphorylation which may have further contributed to an increased inhibitory effect of PLN on SERCA2a [55]. Figure 1B summarizes possible alterations in PLN/SERCA2a microdomain in disease.

Delays in cytosolic calcium reuptake into the SR have also been reported in pre-T2D rat models; however, these changes were not accompanied by altered expression ratios of total PLN:SERCA2a protein [56,57]. For example, in rats fed a high sucrose diet that induced a pre-T2D phenotype (hyperinsulinemia, mild hyperglycemia, and hyperlipidemia) with diastolic dysfunction, a reduction in SR calcium reuptake by ~30% was observed along with significant reductions in both PKA and CaMKII mediated phosphorylation of PLN, yet no changes were observed in total PLN or SERCA2a protein levels, suggesting the decreased SERCA2a activity was driven by upstream alterations in PLN phosphorylation [57]. Consistent with this finding, high-fat-diet-induced obesity in rats resulted in lower levels of PLN Ser16 phosphorylation accompanied by dysregulated calcium handling [58]. However, in contrast, rats given a low dose of Streptozotocin (STZ) and a high fat diet which induced an early T2D phenotype, exhibited an increased phosphorylation of PLN at Ser16, whereas PLN phosphorylation at the CaMKII site was decreased [59]. Additionally, in another study employing a high sucrose-induced pre-T2D insulin-resistant phenotype in rats, calcium reuptake into the SR was prolonged by ~25% compared to that in insulin sensitive cardiomyocytes; however, phosphorylation of PLN at Ser16 along with total PLN and SERCA2a protein levels remained unchanged [56]. Therefore, these data suggest a greater complexity to altered SERCA2a activity and SR calcium reuptake in T2D than that which can solely be explained by reduced PLN phosphorylation.

Indeed, similar observations regarding delayed calcium reuptake into the SR have been observed in cardiomyocytes isolated from MetS rats and from mice fed a high fat diet, with no changes in the PLN phosphorylation status, potential mechanisms were instead attributed to oxidative stress-induced oxidation of SERCA2a and reduced SERCA2a protein levels [60,61]. Interestingly, alterations in PLN Ser16 phosphorylation do not necessarily result in impairments in calcium handling, as a high fat diet in male Wistar rats which induced diastolic dysfunction was associated with reduced PLN phosphorylation at Ser16, in the absence of any clear defects in calcium cycling and reuptake into the SR [62]. In saying that, the therapeutic advantage of maintaining normal PLN phosphorylation in T2D has been demonstrated in the past. For example, in hyperglycemia-induced cardiac hypertrophy, activation of a G-protein coupled receptor TGR5 was shown to improve cardiac function by preventing high glucose-induced decreases in SERCA2a expression and promoting PKA-mediated PLN phosphorylation at Ser16 and subsequent SR calcium reuptake [63]. While this study unfortunately did not assess diastolic dysfunction, it highlights the importance of maintaining normal PKA mediated PLN phosphorylation in regulating SERCA2a mediated calcium reuptake in hyperglycemia.

Considering how alterations in PDE activity may dysregulate SERCA2a activity, it was reported recently that hyperinsulinemia induces systolic and diastolic dysfunction by increasing expression of PDE4D, which contributes to reduced PKA mediated phosphorylation of PLN [64]. Additionally, in response to a high-carbohydrate-diet-induced metabolic syndrome in rats, elevated PDE3 and PDE4 expression was associated with a decreased phosphorylation of PLN and altered calcium homeostasis [65]. However, a limitation of these studies was that assessments of PDEs were made at the whole cell lysate/tissue level. In the future, it would be interesting to further uncover how PDE activities specifically within the PLN/SERCA2a domain is altered in HFpEF. For example, another potential mechanism of decreased SERCA2a activity, may be due to increased PDE activities within the SERCA2a microdomain, leading to decreased cAMP levels and PLN phosphorylation.

## 7. Conclusions

There is still a long way to go before we can fully decipher and understand the precise remodeling of the PLN/SERCA2a domain in HFpEF caused by obesity and T2D. The variable findings from past studies have made it difficult to accurately pinpoint the distinct alterations within the PLN/SERCA2a microdomain that promote diastolic dysfunction (see Figure 1B). However, these studies provide key insights into the likely evolutionary nature of changes in PLN/SERCA2a expression and activity that may depend on the stage and severity of obesity and T2D-induced HFpEF. Furthermore, by compiling a summary of these past literature we have been able to identify key gaps of knowledge that are needed for us to understand the significance of PLN/SERCA2a microdomain remodeling in HFpEF. For example, the main limitation of past studies has been the use of whole cell/tissue lysates in an attempt to explain alterations specifically observed with SERCA2a calcium handling and an overall lack of understanding of cAMP-mediated regulation of PKA and PLN within this microdomain. In future work, the application of highly sensitive FRET cAMP biosensors, such as the Epac1-PLN biosensor, applied to clinically relevant HFpEF models, will allow us for the first time to truly dissect specific alterations in cAMP dynamics that regulate SERCA2a activity. Such as in elucidating any alterations in cAMP concentrations within this microdomain and in β-AR subtype control of cAMP pools and PDE coupling. Ultimately, this knowledge will be imperative in not only our understanding of the pathophysiology of HFpEF induced by obesity and T2D, but also in our attempts to develop effective therapies to treat HFpEF.

## Data Availability

Not applicable.
